# Role of the parahippocampal cortex in memory for the configuration but not the identity of objects: converging evidence from patients with selective thermal lesions and fMRI

**DOI:** 10.3389/fnhum.2015.00431

**Published:** 2015-08-03

**Authors:** Véronique D. Bohbot, John J. B. Allen, Alain Dagher, Serge O. Dumoulin, Alan C. Evans, Michael Petrides, Miroslav Kalina, Katerina Stepankova, Lynn Nadel

**Affiliations:** ^1^Department of Psychiatry, Douglas Mental Health University Institute, McGill UniversityMontreal, QC, Canada; ^2^Department of Psychology, University of ArizonaTucson, AZ, USA; ^3^McConnell Brain Imaging Centre, Montreal Neurological Institute, McGill UniversityMontreal, QC, Canada; ^4^Department of Experimental Psychology, Helmholtz Institute, Utrecht UniversityUtrecht, Netherlands; ^5^Neuropsychology and Cognitive Neuroscience Unit, Montreal Neurological Institute, McGill UniversityMontreal, QC, Canada; ^6^Department of Neurology, Hospital Na HomolcePrague, Czech Republic; ^7^Department of Neurology, First Faculty of Medicine, Charles UniversityPrague, Czech Republic; ^8^ARL Division of Neural Systems, Memory and Aging, University of ArizonaTucson, AZ, USA

**Keywords:** parahippocampal gyrus, hippocampus, human, spatial, location

## Abstract

The parahippocampal cortex and hippocampus are brain structures known to be involved in memory. However, the unique contribution of the parahippocampal cortex remains unclear. The current study investigates memory for object identity and memory of the configuration of objects in patients with small thermo-coagulation lesions to the hippocampus or the parahippocampal cortex. Results showed that in contrast to control participants and patients with damage to the hippocampus leaving the parahippocampal cortex intact, patients with lesions that included the right parahippocampal cortex (RPH) were severely impaired on a task that required learning the spatial configuration of objects on a computer screen; these patients, however, were not impaired at learning the identity of objects. Conversely, we found that patients with lesions to the right hippocampus (RH) or left hippocampus (LH), sparing the parahippocampal cortex, performed just as well as the control participants. Furthermore, they were not impaired on the object identity task. In the functional Magnetic Resonance Imaging (fMRI) experiment, healthy young adults performed the same tasks. Consistent with the findings of the lesion study, the fMRI results showed significant activity in the RPH in the memory for the spatial configuration condition, but not memory for object identity. Furthermore, the pattern of fMRI activity measured in the baseline control conditions decreased specifically in the parahippocampal cortex as a result of the experimental task, providing evidence for task specific repetition suppression. In summary, while our previous studies demonstrated that the hippocampus is critical to the construction of a cognitive map, both the lesion and fMRI studies have shown an involvement of the RPH for learning spatial configurations of objects but not object identity, and that this takes place independent of the hippocampus.

## Introduction

The hippocampal region has been implicated in memory for various kinds of information, such as memory for spatial relations (O’Keefe and Nadel, 1978; Gaffan, 1992), object location (Smith and Milner, 1989), facts and events (Squire, 1992), episodes (Vargha-Khadem et al., 1997; Tulving and Markowitsch, 1998) and establishing stimulus-stimulus relationships (Petrides, 1985; Eichenbaum, 2001). It has also been shown that bilateral lesions to the medial temporal lobe lead to profound memory deficits (Scoville and Milner, 1957; Milner, 1972; Corkin, 1984), while unilateral lesions lead to milder memory impairments that can be detected with cognitive assessment tools in the laboratory (Milner, 1972; Petrides, 1985).

It was only later that the mnemonic role of medial temporal lobe structures adjacent to the hippocampus, such as the perirhinal and parahippocampal cortical regions, has been dissociated from that of the hippocampus in monkeys (Meunier et al., 1993; Murray and Mishkin, 1998; Malkova et al., 2001; Malkova and Mishkin, 2003). Studies showing that patients with lesions involving the parahippocampal cortex are impaired on a memory task do not provide information about the specific role of this structure since the hippocampus receives afferents from the parahippocampal cortex, via the entorhinal cortex (Van Hoesen, 1982; Suzuki and Amaral, 1994) and also directly (Rockland and Van Hoesen, 1994). Thus, impairments after parahippocampal lesions can be attributed to a functional de-afferentation of the hippocampus. On the other hand, the parahippocampal cortex maintains its own strong afferent and efferent connectivity with several cortical areas, including the inferior parietal cortex (Van Hoesen, 1982; Blatt et al., 2003). In order to show that the parahippocampal cortex is itself critical in certain aspects of mnemonic processing, it is necessary to show that patients with lesions to the hippocampus alone are either not impaired on particular tasks, or impaired less than patients with lesions to the parahippocampal cortex. Such a result would argue against the notion that deficits after parahippocampal damage merely reflect a functional hippocampal lesion.

In the human brain, lesions to parahippocampal cortex and cortical regions providing input to it, such as the lingual gyrus and the inferior parietal cortex have been implicated in topographical memory loss (Landis et al., 1986; Habib and Sirigu, 1987; Hublet and Demeurisse, 1992; Maguire et al., 1996; Aguirre and D’Esposito, 1999; Epstein et al., 2001), i.e., an impairment in the ability to find one’s way in the environment. Although studies of topographical amnesia point to damage in the parahippocampal cortex, or to regions surrounding it, they do not exclude the possibility of a functional hippocampal lesion.

Very few case studies have effectively dissociated the mnemonic role of the parahippocampal cortex from that of the hippocampus with selective lesions (Ploner et al., 2000). In a study of spatial memory by Bohbot et al. (1998), epilepsy patients with selective thermal lesions to the right hippocampus (RH) were not impaired at finding a sensor hidden under a floor carpet, relative to patient control participants, after a 30-min delay while using novel starting positions, thereby requiring allocentric spatial memory, i.e., navigation that is independent of the position of the observer. On the other hand, patients with lesions to the parahippocampal cortex were severely impaired on this spatial task relative to the control group. Interestingly, patients and controls exhibited similar search patterns on the first trial, indicating that planning a search for the target is not dependent on medial temporal lobe areas (Bohbot et al., 2002). In monkeys, lesions to the parahippocampal cortex, but not the hippocampus, impaired performance on a delayed match-to-sample task that required memory for the locations of two objects presented over two of three foodwells (Malkova and Mishkin, 2003). In this paradigm (Parkinson et al., 1988), monkeys are shown the locations of two objects in a sample phase, and in a subsequent test phase, they are shown two objects identical to one of the previously seen objects. In order to receive a reward, the monkey must select the object at the same location it occupied in the sample phase.

Interestingly, a functional Magnetic Resonance Imaging (fMRI) study using navigation tasks that required different types of spatial representations demonstrated that, although the medial temporal lobe was activated on all tasks, the core of the activity was in the posterior parahippocampal gyrus with minimal involvement of the hippocampus itself (Rosenbaum et al., 2004). Several other fMRI studies have also demonstrated a clear dissociation between the function of the hippocampus and the parahippocampal cortex. In a virtual navigation task in which participants had to navigate in an environment devoid of landmarks during fMRI scanning and, therefore, were forced to use an egocentric navigation strategy, bilateral activity was observed in the parahippocampal cortex but not the hippocampus (Weniger et al., 2010). Morgan et al. (2011) had participants view pictures of familiar landmarks while lying in an fMRI scanner. Activity in the hippocampus was related to the distance between the landmarks, while activity in the parahippocampal cortex was related to landmark repetition. In an fMRI study by Howard et al. (2011) in which subjects were administered an incidental target detection task, the hippocampus was selectively active when the spatial relationships between the objects and the background context changed. In contrast, the parahippocampal cortex was selectively active for novel scenes. In Hartley et al. (2003), the authors distinguish between performance-independent effects and performance-related effects. They report activity in the parahippocampal cortex during the wayfinding task, unrelated to performance. In contrast, they report activity in the hippocampus during wayfinding that is associated with accurate performance. Our previous studies also demonstrated that the hippocampus is critical to learning the spatial relationships between landmarks in the environments (Bohbot et al., 2004; Konishi et al., 2013). Perhaps a critical element that distinguished studies that showed a critical involvement of the hippocampus vs. parahippocampal cortex in allocentric spatial memory, is that the hippocampus seemed to require a “construction” process, from memory, of detailed relationships between objects or landmarks in the environment, in a scene or episodes (Rosenbaum et al., 2009). Taken together, these studies and several others (Duzel et al., 2003; Goh et al., 2004; Pihlajamäki et al., 2004; Köhler et al., 2005) further support the notion that the parahippocampal cortex is functionally dissociable from the hippocampus. Still, very few studies have dissociated the role of the parahippocampal cortex from that of the hippocampus in brain damaged patients, because selective lesions to the hippocampus rarely occur as a result of vascular incidents, diseases, accidents, or surgical interventions.

While there are many fMRI studies and many lesion studies looking at spatial memory and the medial temporal lobe, there are very few reports combining lesion and fMRI using the same paradigm. In the present article, we report a dissociation of the role of the parahippocampal cortex from that of the hippocampus in memory for the configuration of objects but not their identity. This was achieved with a special cohort of patients with small selective thermocoagulation lesions to the hippocampus and/or the parahippocampal cortex in an attempt to alleviate epilepsy. Our results from the patient study were then confirmed with a second study involving fMRI with healthy young adults.

## Experiment 1: Cognitive Lesion Study

### Materials and Methods

#### Participants

Two control groups and four groups of brain-operated patients were tested (see Table 1). These patients have been described elsewhere (Bohbot et al., 1998). One control group consisted of patients with back-pain problems and no epileptic problems. The second control group consisted of patients with epilepsy who had not undergone brain surgery. Of the two control groups, the patients with epilepsy control group is similar to the experimental groups with respect to the type of disorder and medication taken by patients and, therefore, serves as a better control. The study was approved by the institutional review board and informed consent was obtained from all participants in accordance to the guidelines of the local ethics committee.

**Table 1:** **Demographic and neuropsychological logical characteristics of the participants in the brain lesion study**.

Group	Sex		Age		Wechsler IQ		Wechsler memory scale	
	M	F	Mean	Range	Mean	Range	Mean	Range
**Brain-operator groups**								
Right Hippocampal	3	2	35.6	29–42	109.8	96–131	107.8	89–126
Right Parahippocampal	3	2	45	38–59	94	82–105	102	81–129
Left Hippocampal	0	3	41.7	37–50	90.6	87–96	96.3	89–103
Left Parahippocampal	1	0	34	–	99	–	87	–
**Control groups**								
Back-Pain Patient Control	5	3	41.4	29–57	119	96–133	126	98–143
Epileptic Patient Control	5	5	26.5	17–43	99.3	80–129	107.1	99–143

##### Back-Pain control group

Eight patients living with chronic lumbar back-pain were selected as control subjects as they suffered from a chronic medical condition that does not directly affect the central nervous system.

##### Epilepsy control group

Ten patients with epilepsy who had not undergone a neurosurgical procedure, nor thermal lesion were used as controls. These patients were on non-toxic AntiEpileptic Drug (AED) therapy at the time of the study, similar to the medication received by the brain operated patients, the difference being that the purpose was to control their epilepsy. None of the patients included presented clinical symptoms of medication toxicity. The antiepileptic drug therapy included one, two or three of the following: carbamazepine, primidone, valprolate, phenytoin, clonazepam, lamotrigine, vigabatrin, and barbiturate. The patients’ presentations were not affected by seizures on the day of testing.

##### Brain-operated groups

Fourteen patients who underwent brain surgery in an attempt to alleviate refractory epilepsy are included here. The following exclusion criteria was used: Wechsler IQs below 75, psychiatric disorders, gross brain atrophy and left-handedness. Patients were tested 4–17 years after surgery. At the time of testing, all patients were on an anticonvulsant similar to those taken by the epilepsy control group. None of the patients presented any symptoms of drug toxicity (as assessed by a neurologist) and no patient had clinical or Electroencephalogram (EEG) seizures on the day of testing.

The anatomical landmarks used to identify patients’ lesions have been outlined elsewhere (Bohbot et al., 1998). Patients with lesions were divided into two groups according to whether or not they had damage to the parahippocampal cortex. Hippocampal lesions include damage to the hippocampus proper, as well as the dentate gyrus, and the subicular complex. Damage to the parahippocampal cortex is characterized by damage to the posterior parahippocampal gyrus, the neo-cortical region posterior to the entorhinal cortex and perirhinal cortex.

##### Right hippocampus

**Figure 1:**
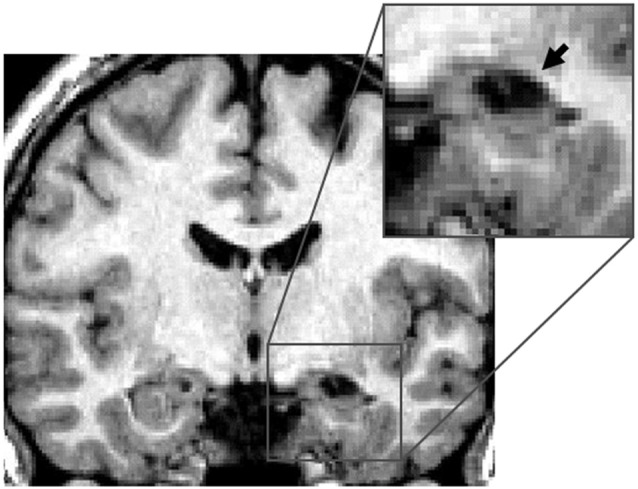
**Right hippocampal lesion**. MRI section in a coronal plane, zooming-in on the lesion to the right hippocampus (RH) of patient KJ (indicated with the arrow). This section was taken 12 mm posterior to the anterior commissure (*Y* = −12) in Talairach standard stereotaxic space (Talairach and Tournoux, 1988).

This group consisted of five patients who had damage to the RH. Patient KJ had damage to the anterior portion of the hippocampus (Figure 1) and to the amygdala, and minor damage to the perirhinal cortex. Patient BS had a lesion to the right anterior and posterior parts of hippocampus, some damage to the right amygdala, and minor damage to the anterior portion of the right perirhinal cortex and the right inferior temporal neocortex. Patient FL had bilateral damage to the amygdala and damage to the right anterior hippocampus. Patient KP had a right hippocampal lesion, specifically damage to the anterior and posterior parts of the hippocampus and additional damage to the right amygdala only. Finally, Patient MJ had a right anterior lesion to the hippocampus with additional damage to the right amygdala only. None of the patients included in this group had any damage to the parahippocampal cortex.

##### Right parahippocampal cortex

**Figure 2:**
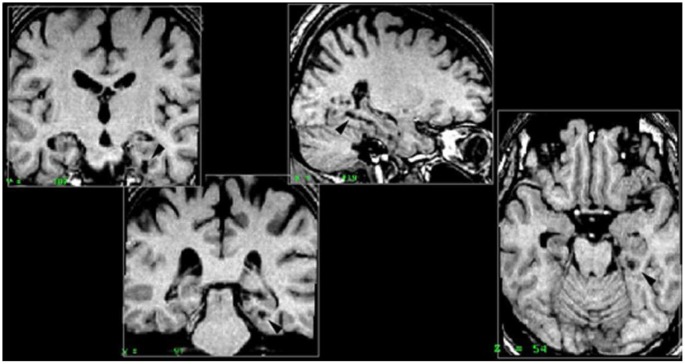
**Right parahippocampal lesion**. MRI section in a coronal plane, zooming-in on the lesion to the right parahippocampal cortex (RPH) of patient PP (indicated with the arrow). This section was taken 30 mm posterior to the anterior commissure (*Y* = −30) in Talairach standard stereotaxic space (Talairach and Tournoux, 1988).

All five patients included in this group had damage to the right posterior parahippocampal cortex. Patient PP also had complete damage to the anterior part of the hippocampus, and partial damage to the following regions: posterior part of the hippocampus, amygdala, perirhinal and entorhinal cortices. Patient MJa had damage to both the anterior and posterior hippocampus, partial bilateral (but not symmetrical) damage to the amygdala and the entorhinal and perirhinal cortices were intact. PM had damage to the right parahippocampal cortex (RPH), anterior and posterior portions of the RH, the right amygdala, but no damage to the perirhinal cortex or entorhinal cortex. Patient PV had damage to the right anterior hippocampus and the right perirhinal cortex. Patient KrA had damage to the parahippocampal cortex, entorhinal cortex, and perirhinal cortex, but the RH was intact (Figure 2).

##### Left hippocampus

Three patients, KS, SV, and VP, with lesions to the left hippocampus (LH) were included in this group, Patient KS presented with lesions in the left anterior hippocampus, left amygdala, and minor damage to the left entorhinal cortex and left perirhinal cortex. Patient SV had damage to the LH (posterior region), bilateral damage to the amygdala, and damage to the anterior portion of the left perirhinal cortex. Patient VP had a lesion to the left anterior and posterior regions of the hippocampus and the left amygdala was partially damaged. None of the patients included in this group presented with any damage to the parahippocampal cortex.

##### Left parahippocampal cortex

One patient, SI, had damage to the left parahippocampal cortex (LPH), as well as some damage to the left amygdala and left perirhinal cortex.

#### Procedure

**Figure 3:**
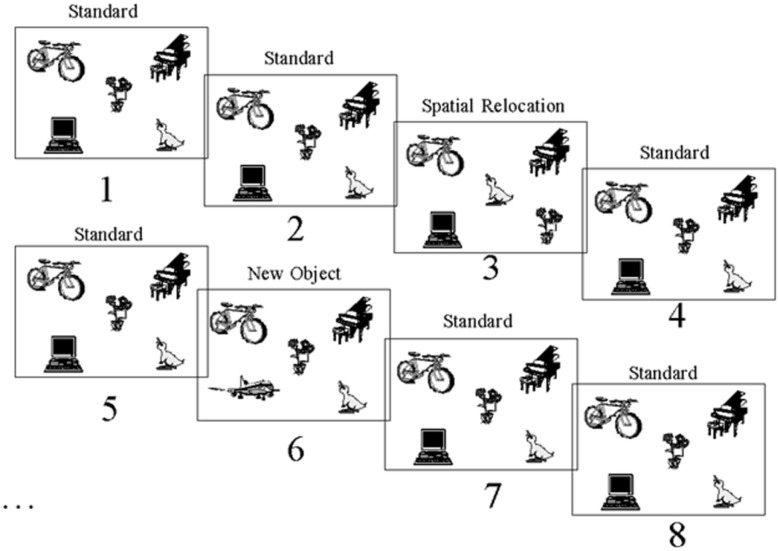
**Stimuli**. Example of one of the two sets of stimuli used showing the standard scene, spatial configuration change and object identity change. This stimulus set was used for either the spatial or object task, and another set of stimuli was used in the other task.

Computerized tasks were developed to assess patients’ for two types of object information memory: changes in the spatial configuration of objects, and changes in the objects’ identity. These computerized tasks were designed according to the oddball fashion for use with evoked potentials (Allen et al., 1994). The tasks were as follows: a standard display depicting five unrelated objects appeared on 80% of the trials (standards), and this standard display was altered on 20% of the trials (Figure 3). A new object appeared in place of one of the objects on the standard display on 10% of the trials (object identity change). On another 10% of the trials, the location of two of the objects from the standard display were switched (spatial configuration change).

Participants were asked to respond to spatial configuration changes on the spatial task (targets) and ignore the object identity changes (distractors). In the object task, participants were tested with a different set of stimuli and asked to respond to a change in object identity (target), while ignoring the changes in spatial configuration (distractors). Two sets of similar stimuli were developed. Each stimulus set could be used in either task, but for any given participant each stimulus set was used for only one task. The order of task presentation was counterbalanced.

In both the object and spatial tasks, five blocks of 50 displays of objects were presented. Each block of 50 was comprised of five sets of 10 displays, such that only one object change and one spatial configuration change occurred within each set of 10 displays. Each display was presented for a duration of 1496 ms, followed by a black screen for 2000 ms before the onset of the next display. For each trial, the participant’s reaction time was recorded up to a maximum of 1500 ms.

##### Spatial task

Participants were instructed to press the left shift key when the standard scene was presented (occurred on 80% of the trials). When two of the five objects exchanged positions (spatial configuration change; occurring on 10% of trials), participants were instructed to press the right shift key. During this task, one of the objects was replaced by a novel object on another 10% of the trials (object identity change). Participants were instructed to ignore this object change and to press the left key. The keyboard was marked with the word “space” on the right, and the word “object” on the left in order to cue the participants.

##### Object task

Participants were instructed to press the left shift key when the standard scene was presented (80% of the trials). When one of the five objects was replaced by a novel object (object identity change), which happened 10% of the time, participants were instructed to press the right key. During this task, an exchange of the position of two of the objects (spatial configuration change) occurred on another 10% of the trials. The participants were instructed to ignore this spatial configuration change and to press the left key. The keyboard was marked with the word “object” on the right, and the word “space” on the left in order to cue the participants.

In summary, during either task, participants indicated their response by either pressing the left key for standards and distractors (“NO” response), or a right key for the targets (“YES” response). The participant’s target detection was “correct” if the right key was pressed for the change in object identity during the object task, or if the right key was pressed for the change in configuration of objects in the spatial task. A response was incorrect if the right key was pressed for either the standards or irrelevant changes.

#### Analysis

A non-parametric analysis of variance, the Kruskal-Wallis H test, was used to analyze the data as the assumption of a normal distribution cannot be made with small groups. The single patient with a left parahippocampal lesion was not included in any of the statistical analyses. One participant from the Epilepsy patient control (EPC) and one participant from the RH group (patient BS) were outside the distribution of the number of non-responses to standards, and over two standard deviations from the mean. The high number of non-responses to the standard situation was an indication that they were not participating in the task; they were therefore excluded from the analysis. Responses to the standard displays that followed the identity or spatial switch events were never included in the analyses, as these represented a change back to the standard condition. The five groups included in the analyses were: the back-pain control participants (BPC), the EPC participants, the RH, RPH, and LH groups. For both the object and spatial detection task, the Kruskal-Wallis test was performed on the correct “YES” responses divided by the total number of responses made (either to spatial or object changes). Statistical analyses were also performed on non-responses, “NO” responses to standard scenes, and slowing latencies to irrelevant changes. Further analysis was done with the Wilcoxon Rank Sum Test for comparing two independent samples (two-tailed test). First, we compared the Back-Pain Control group to the Epilepsy Patient Control group. The Back-Pain Control group and the Epilepsy Patient Control group were then compared with each surgical patient group, and the group with lesions to the RH was compared to the group with lesions to the RPH.

### Results

On average, participants responded to 97.5% of the stimuli within the allotted time, and this rate of response did not differ by group for either the spatial task (Kruskal-Wallis rank test, *H* = 8.24, df = 4, n.s.) or the object task (Kruskal-Wallis rank test, *H* = 1.41, df = 4, n.s.). During both the object and spatial tasks, only the trials during which participants made a response were analyzed. Consequently, all the “YES” and “NO” responses added to 100% of the analyzed trials. A comparison of the five groups showed that participants’ correct (“NO”) responses did not differ on standard scenes of the spatial task (Kruskal-Wallis rank test, *H* = 2.63, df = 4, n.s.) or the object task (Kruskal-Wallis rank test, *H* = 4.33, df = 4, n.s.), and since these are not of primary interest, they will not be discussed further. There were no group differences for the reaction times to object identity changes during the spatial task (Kruskal-Wallis rank test, *H* = 0.50, df = 4, n.s.) or to spatial configuration changes during the object task (Kruskal-Wallis rank test, *H* = 5.67, df = 4, n.s.).

#### Spatial Task

**Figure 4:**
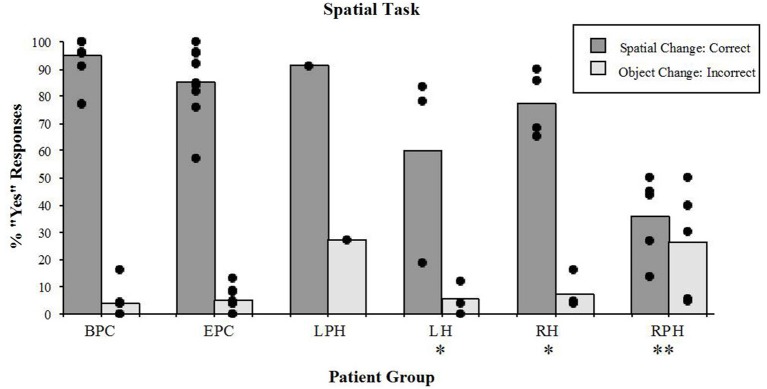
**Spatial task**. Percent scores of correct detection of the spatial configuration change (target), and incorrect detection of the irrelevant object identity change (distractor). Each bar represents the mean of a group. The scores of individual participants for each group are also displayed. BPC: Back-Pain Control, EPC: Epilepsy Patient Control, LPH: Left Parahippocampal Cortex, LH: Left hippocampus, RPH: Right Parahippocampal Cortex, RH: Right Hippocampus. *Significantly different from the BPC group in responses to spatial changes (*P* < 0.05). **In responses to spatial changes, significantly different from the BPC and EPC groups (*P* < 0.005) and from the RH group (*P* < 0.05); in responses to the object changes, significantly different from the BPC (*P* < 0.01) and EPC (*P* < 0.05) groups.

The right parahippocampal patients, and to some extent the right and LH patients showed poor discrimination of the spatial configuration from the object identity changes in the spatial task (Figure 4). In other words, patients with damage to the parahippocampal cortex answered “YES” to any change, whether spatial or object changes, showing no discrimination between the two. On the other hand, patients with damage to the RH and LH correctly responded “YES” more often to the spatial change, than to the object change. The detection of spatial configuration change (i.e., the “YES” response) was different across groups (Kruskal-Wallis rank test, *H* = 17.71, df = 4, *p* < 0.001). The Wilcoxon Rank Sum Test for two independent samples showed that performance of the two control groups did not differ significantly. The right parahippocampal participants were impaired relative to the control participants with epilepsy (*z* = 2.93, *p* < 0.005), and relative control participants with back-pain (*z* = 2.89, *p* < 0.005). The left and right hippocampal patients were impaired relative to the BPC (left: *z* = 1.98, *p* < 0.05; right: *z* = 2.33, *p* < 0.05); however, they were not impaired relative to the epileptic patient control participants (left: *z* = 1.48, n.s.; right: *z* = 0.85, n.s.). This implies that the left or right hippocampal lesion itself did not significantly increase the impairment resulting from the change in performance observed in participants with epilepsy. The lesion that included the RPH, however, did impair performance on this task. In addition, the group with lesions to the RPH was significantly impaired relative to the RH lesion group (*z* = 2.33, *p* < 0.05), thus showing that the impairment resulting from the RPH was dissociated from that of the RH. These results were previously reported elsewhere (Bohbot et al., 2000).

While participants were engaged in the spatial task, there were significant differences (Kruskal-Wallis rank test, *H* = 9.76, df = 4, *p* < 0.05) in the number of incorrect “YES” responses to the object identity change (distractors; Figure 4). The Wilcoxon Rank Sum Test showed that only patients with damage to the RPH were impaired relative to the patient control group with epilepsy (*z* = 2.15, *p* < 0.05) and relative to the control group with back-pain (*z* = 2.59, *p* < 0.01). None of the other tested comparisons were significant. These results show that only the patients with lesions to the RPH were affected by the presence of distractors in the spatial task.

#### Object Task

**Figure 5:**
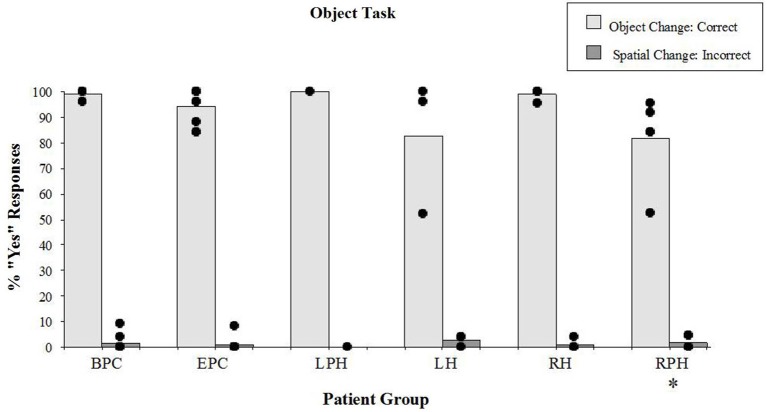
**Object task**. Percent scores of correct detection of the object identity change (target), and incorrect detection of the spatial irrelevant change (distractor). Each bar represents the mean of a group. The scores of individual participants for each group are displayed. See legend of Figure 4 for the description of labels. *Significantly different from the BPC (*p* < 0.01) and the RH (*p* < 0.05) groups in responses to object changes.

There were significant differences in correct detection of the object identity change (“YES” responses) during the object task (Figure 5) across all groups (Kruskal-Wallis rank test, *H* = 11.91, df = 4, *p* < 0.01). The Wilcoxon Rank Sum Test showed that the two control groups were not different from each other and the right parahippocampal group was impaired relative to the back-pain control group (*z* = 3.01, *p* < 0.01) but not compared to the control participants with epilepsy (*z* = 1.91, n.s.). Patients with lesions to the RH or LH were not impaired on this task, either relative to the BPC or relative to the control participants with epilepsy. However, patients with lesions to the RPH were impaired relative to patients with right sided lesions to the hippocampus (*z* = 2.38, *p* < 0.05) suggesting that the right parahippocampal lesion itself was responsible for the impairment. It should be noted, however, that the performance of all but one participant in the right parahippocampal group ranged between 84% and 96% correct (see Figure 5) on the object identity task, which is in striking contrast to performance on the spatial configural task where the same patients scored an average of 35% correct, and thus the noted impairment has a small impact on the actual performance.

There were no differences (Kruskal-Wallis rank test, *H* = 3.03, df = 4, n.s.) in incorrect “YES” responses to the irrelevant spatial configuration change (distractors; see Figure 5). It should be noted that patients with lesions to the RPH were not affected by the distractors during the object task, which contrasts with the fact that they were severely affected by the distractors while performing the spatial task. These results show that the patients with lesions to the RPH were selectively impaired at the spatial task, with preserved performance in the object task.

### Discussion

The present experiment measured memory for object identity (learning which objects were part of a standard scene) and memory for the configuration of objects (learning the spatial arrangement of objects on the screen). Both of these tasks required participants to react to one kind of change and ignore the other, in order to avoid responses due solely to novelty.

Patients with lesions to the RPH were impaired on the spatial task even when compared with epilepsy control participants and participants with lesions to the RH. They were largely unable to detect the spatial configuration changes, and responded equally to both the spatial configuration and object identity changes (Figure 4). The fact that patients with lesions to the RPH were impaired relative to patients with lesions to the RH indicates that the deficit resulting from the lesion in the parahippocampal cortex is unlikely simply to reflect a functional lesion in hippocampus. Thus, it suggests that the parahippocampal cortex plays a role in this spatial memory function above and beyond transmitting information to the hippocampus.

Patients with lesions to the RH or LH were impaired relative to the back-pain control group, but not relative to the epilepsy control group on the spatial task. Despite their impairment, the patients with lesions to the hippocampus performed well (average 77% correct for the right and 60% correct for the left) compared to with patients in the right parahippocampal group (average 35% correct). A table-top task that assesses similar functions as our spatial task, was administered by Watson et al. (2013) to patients with lesions to the hippocampus. In this study, object was placed on a table-top and participants were asked to remember where the objects are located. After a 4 s blind delay, participants were asked to replace the objects in the correct relative positions. Patients with lesions to the hippocampus made significantly more “swap” errors, in which they often swapped the relative position of objects. At first, these findings appear inconsistent with the current study, in which patients with lesions to the hippocampus were not impaired at indicating when the spatial location of two objects was swapped. There are two possible explanations for this discrepancy. The first is that in Watson et al. (2013), the lesions were not specific to the hippocampus and the authors report that all three patients had varying degrees of damage to the temporal lobes. Although it is not specified in the study, it is highly likely that this also includes damage to the parahippocampal cortex. Therefore, the “swap” errors observed in Watson et al. (2013) may in part be related to damage to the parahippocampal cortex. The second reason for the discrepancy may be that in Watson et al. (2013), the comparison group was healthy controls, while in the current study, the comparison groups were other patients. If Watson et al. (2013) compared their sample to other patient groups, then their impairments may be less pronounced. The third is that, in Watson et al. (2013) the location of objects has to be reconstructed from memory, whereas in the current study, there is no “reconstruction” required by the patients. Instead, patients are presented with images of objects, and they need to recognize when a swap has taken place. This interpretation is consistent with a paper by Stepankova et al. (2004) where patients with selective lesions to the hippocampus were impaired at reconstructing from memory the location of objects. In that 2004 study, patients were impaired despite the fact that swaps were taken into account, providing evidence for the role of the hippocampus in the construction of a cognitive map, of object locations in this case. In the current study, the significance in the comparison of the hippocampal groups in relation to the control group with back-pain indicates that the impairment is the result of factors related to both having epilepsy (i.e., medication, disorder, dysfunction of the medial temporal lobe etc.) and the thermal lesion. However, since there were no differences between the two groups with hippocampal lesions and the epilepsy control group, ascribing a possible deficit to the specific thermal lesion within the medial temporal region is not possible. We can therefore conclude that medial temporal areas, including the LH and RH are involved in this task, and that the remaining ability of the hippocampal groups, in the performance of the spatial task, can be accounted for by their intact parahippocampal cortex.

All of the patients (with RH or LH or parahippocampal cortex lesions) had comparable scores on the object task (Figure 5). If participants with thermal lesions to the RPH were poor at learning the spatial configuration of the objects, it is understandable that they successfully ignored these changes while performing the object task. The fact that they performed well on the object task (average 82% correct) compared with the spatial task (average 35% correct) indicates that they encoded the identity of objects during the task. Our data show that the deficits in patients with lesions to the RPH were severe for the spatial configuration but not for object identity.

## Experiment 2: fMRI with Healthy Young Adults

### Materials and Methods

#### Participants

Eight healthy young adults (mean age = 31.75 ± 5.00; four women and four men) participated in the fMRI experiment. All participants were right handed and had normal vision. None of the participants had any history of neurological or psychiatric illnesses. Recruitment was done by word of mouth. The study was approved by the institutional review board and informed consent was obtained from all participants in accordance to the guidelines of the local ethics committee.

#### Procedure

##### Cognitive task

Participants were presented with five objects on a projection screen; a new set of similar objects was used, relative to those presented in the patient study. On 10% of the trials, participants viewed a change in spatial locations of two of the objects (spatial configural change) and on another 10% of the trials one object were replaced by a new one (object identity change). For each set of five objects, the new object always remained the same (a sixth object). In the spatial task, participants were instructed to respond to spatial configuration changes (targets) and ignore the object identity changes (distractors). In the object task, participants were tested with a different set of stimuli and were asked to respond to a change in object identity (target), while ignoring the changes in spatial configuration (distractor). Consequently, participants view the same types of changes in both tasks, but they have to discriminate which changes they must respond to depending on whether they are engaged in the spatial configuration task or the object identity task.

##### Control task

In the control tasks, participants viewed the same objects as in the experimental tasks, but they were instructed to always press the same key so that no change needed to be detected.

##### Neuroimaging

**Figure 6:**
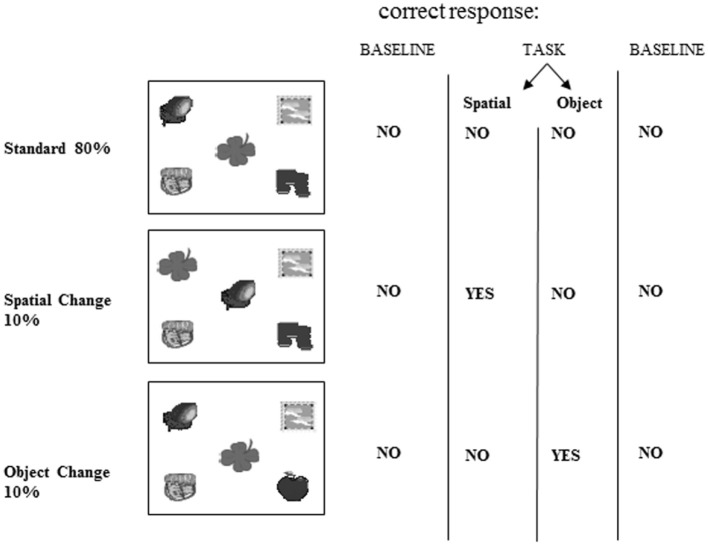
**Overall experimental design of the fMRI oddball task showing the control baseline condition before and after the experimental tasks**.

**Figure 7:**
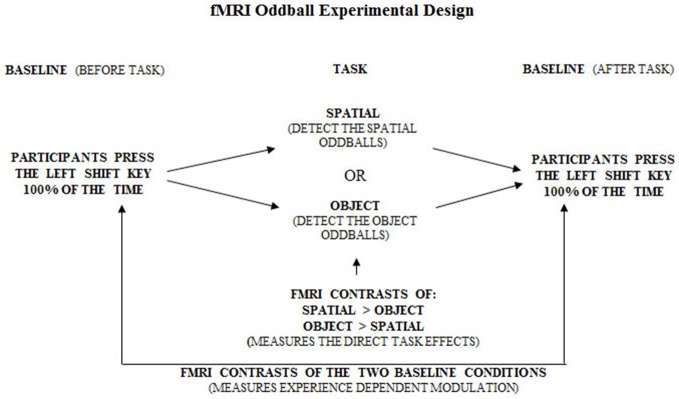
**Overall experimental design of the fMRI oddball task showing the correct response that should be made by participants when viewing the standard condition, spatial and object change “oddball” conditions**.

**Figure 8:**
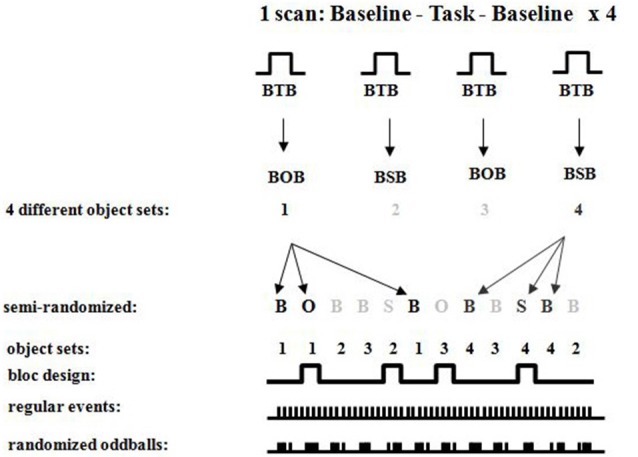
**Detailed fMRI experimental design showing randomized oddballs within a semi-randomized block design showing the different sets of objects used for the different conditions**.

Participants were scanned in a Siemens 1.5 T Scanner at the Montreal Neurological Institute. Participants were comfortably placed in the scanner with their heads immobilized with an air cushion. Prior to the functional scans, T1-weighted anatomical images were acquired to allow coregistration of functional and anatomical data. A three-dimensional gradient echo acquisition was used to collect 80 contiguous 2 mm T1-weighted images in the sagittal plane. Seven whole brain fMRI scans per participant were collected. Each functional scan consisted of 120 T2*-weighted image volumes acquired at 4 s intervals, giving a total duration of 8 min per scan. Each functional scan was acquired using 26 contiguous 5 mm axial slices positioned parallel to the hippocampus and covering the entire brain (TR = 4000 ms; echo *time*_*(TE)*_ = 50 ms; field of view = 320 mm^2^; matrix size = 64 × 64; 120 whole brain acquisitions/run). Blood oxygen level dependent (BOLD) signal images were spatially smoothed (6 mm Gaussian kernel), corrected for motion, and linearly transformed into standard stereotaxic space (Talairach and Tournoux, 1988) using in-house software (Collins et al., 1994). Each scan consisted of four blocs, each having (a) baseline control; (b) object or spatial task; and (c) baseline control, with different sets of stimuli for each of the blocs (see Figures 6–8). During each bloc (the two baseline conditions and the experimental condition) 60 stimuli were presented with 1 s “on” and 1 s “off”, thus a bloc had a duration of 2 min. Twenty-eight sets of stimuli were used, each following the same format so that one set of stimuli was used during only one bloc per subject. Each stimulus set could be used in either task, but for any given participant a stimulus set was used for only one task. The order of task presentation was counterbalanced and the order of bloc segments was randomized. The objects appearing in each of the 28 sets were randomized between participants. The data were analyzed by correlating a predicted hemodynamic response curve with the fMRI time-courses, using FMRISTAT (Worsley et al., 2002). This paradigm allowed us to contrast neuronal activity between the baseline scan and the spatial task or object task, and the two baseline scans before and after the experimental condition. Since the oddball appeared at random intervals within each 20 s period, an event-related fMRI analysis was done by comparing the hemodynamic responses to the object or spatial oddballs when presented as a targets or distractors.

Each block involved visual presentation of a given display of five objects in 80% of the occurrences (standards; see Figure 6). Spatial configural changes from the standard display occurred in 10% of the presentations (i.e., two objects exchanged positions see Figure 6 and object identity changes occurred in 10% of the presentations (a 6th object replaced any one of the five in the display; see Figure 6). During the scans, participants responded to each visual display by 1-always clicking the left mouse button (baseline see Figure 7), 2-detecting the spatial changes with the right mouse button, or 3-detecting the object changes with the right mouse button, clicking on the left for all other non-target stimuli (see Figure 6). Stimuli were viewed for 1 s with 1 s inter-stimulus interval. There were four repetitions of Baseline-Task-Baseline per scan and seven scans per participant (totaling 28 repetitions; Figures 6–8). Each repetition involved a unique set of objects (28 sets of objects). The order of conditions was presented in a semi-random fashion. Participants practiced before entering the scanner. Based on our* a priori* hypothesis, an uncorrected *p*-value of 0.001 was used for voxels in the predicted regions of interest (*N* = 8, *t* = 4.785), namely the parahippocampal cortex which we hypothesized to be involved in the spatial task based on the current experiment with brain lesioned patients, and the temporal and parietal cortices because of their well-known roles in processing information about object identity for the temporal cortex and visuo-spatial perception for the parietal cortex. A statistical *t*-threshold of 4.785 at *p* < 0.001 is similar to other studies that report parahippocampal activity (Goh et al., 2004; Köhler et al., 2005; Weniger et al., 2010; Howard et al., 2011; Morgan et al., 2011). For the whole brain, a Bonferroni correction for multiple comparisons was used to calculate the *t*-statistical threshold at *p* < 0.05 (*N* = 8, *t* = 22.04). All peaks above 4.785 outside our regions of interest are reported, but they are not discussed since they do not cross the threshold for whole brain Bonferroni correction for multiple comparisons. Regions close to the significant threshold for our regions of interest are also identified.

### Results

#### Spatial Task

**Figure 9:**
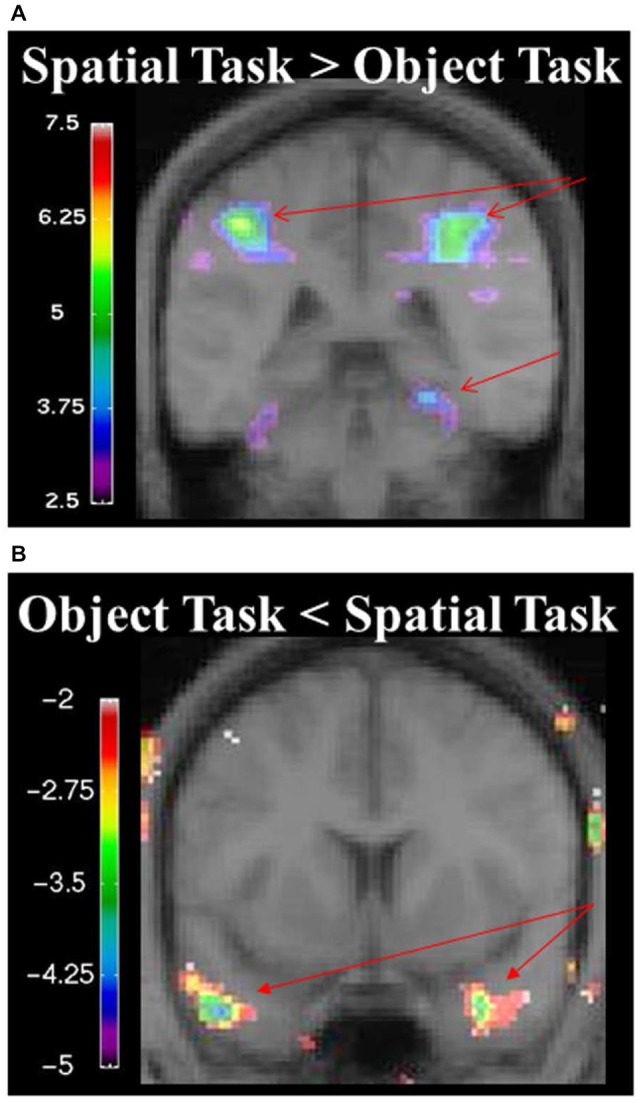
**(A)** fMRI results from the spatial memory configural task contrasted against the object identity task. Results are overlaid on the mean structural image. Hemispheres are indicated by L (left) and R (right). Increase in activity in the RPH (*x* = 24, *y* = −38, *z* = −16; *t* = 3.79, *p* < 0.005), and bilateral activation in the parietal lobes (left: *x* = −26, *y* = −56, *z* = 42; *t* = 8.32, *p* < 0.00005 right: *x* = 26, *y* = −60, *z* = 36; *t* = 8.64, *p* < 0.00005). **(B)** fMRI results from the object identity task contrasted against the spatial memory configural task. Significant increase activity in the bilateral anterior temporal lobe (left: *x* = −46, *y* = 8, *z* = −34; *t* = −4.15, *p* < 0.005; right: *x* = 34, *y* = 0, *z* = −28; *t* = −3.99, *p* < 0.005). The negative values in the object identity task represent greater activity during that task when it was contrasted to the spatial task.

During the spatial memory configural task, there was an increase in activity in the RPH (*x* = 24, *y* = −38, *z* = −16; *t* = 3.79, *p* < 0.005) when this active condition (detection of spatial configural change) was contrasted to the active memory for object identity task (detection of the object identity change) (Figure 9A). This finding is consistent with our original premise as well as the results from the experiment with the brain lesioned patients. There was also bilateral activation in the parietal cortex (left: *x* = −26, *y* = −56, *z* = 42; *t* = 8.32, *p* < 0.00005; right: *x* = 26, *y* = −60, *z* = 36; *t* = 8.64, *p* < 0.00005; Figure 9A) and the right frontal cortex (Table 2). There was no significant difference in fMRI activity in the hippocampus between the spatial memory configural task and the object identity task.

**Table 2:** **fMRI results from the spatial memory configural task contrasted against the object identity task**.

				Coordinates			*t*-stat
				*X*	*Y*	*Z*
Positive	Right	Frontal	Inferior frontal gyrus	32	28	4	5.44
		Temporal	Parahippocampal cortex	24	−38	−16	3.79
		Parietal	Precuneus	2	−50	56	5.12
			Inferior parietal lobe	34	−70	20	5.44
			Superior parietal lobe	26	−60	36	8.64
	Left	Parietal	Precuneus	−4	−70	50	5.07
			Inferior parietal lobe	−36	−58	62	4.96
			Superior parietal lobe	−26	−56	42	8.32
		Occipital	Middle occipital gyrus	−34	−76	24	5.28
Negative	Right	Temporal	Anterior temporal lobe	34	0	−28	−3.99
	Left	Temporal	Anterior temporal lobe	−46	8	−34	−4.15

*All areas showing positive (i.e., increases during the spatial configural task over the object identity task) and negative (i.e., increases during the object identity task over the spatial configural task) differences in activity between the two conditions are listed*.

**Figure 10:**
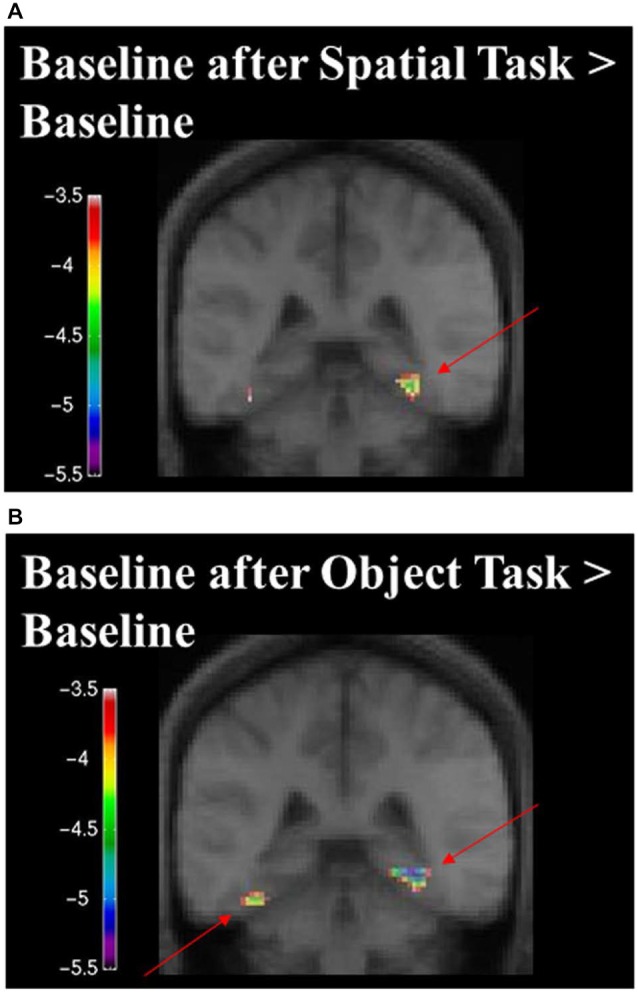
**Contrast of baseline fMRI scans before and after the task for the two conditions showing task specific evidence of repetition suppression in the same areas of the brain required for learning the task**. Results are overlaid on the mean structural image. Hemispheres are indicated by L (left) and R (right). **(A)** fMRI results from the two baseline conditions, before and after the spatial task, contrasted against each other. After the spatial task, there was decreased in activity in the RPH (*x* = 32, *y* = −40, *z* = −16; *t* = −5.15, *p* < 0.001). **(B)** fMRI results from the two baseline conditions, before and after the object task, contrasted against each other. After the object task, there were decreases in activity in the left fusiform (*x* = −38, *y* = −48, *z* = −16; *t* = −6.34, *p* < 0.0005) and right parahippocampal cortex (*x* = 26, *y* = −42, *z* = −10; *t* = −4.72, *p* < 0.005). This is interesting in the light of the fact that participants did not engage in any task during these baseline conditions and that the pattern of change that occurred in the contrast between these baseline conditions was different for the baseline conditions around the spatial task vs. those around the object task. Note that the spatial configuration task and the object identity task were not included in these analyses.

When contrasting the two baseline conditions, before and after the spatial task for that specific set of stimuli, there were decreases in activity in the RPH (Figure 10A; Table 3) (i.e., for the contrast of baseline after the spatial task minus baseline before the spatial task, for a given set of stimuli). This is interesting in the light of the fact that participants did not engage in any task during these baseline conditions and that the pattern of change that occurred in the contrast between these baseline conditions was different for the baseline conditions around the spatial task vs. those around the object task.

**Table 3:** **Contrast of baseline conditions before and after the spatial task showing the decrease in fMRI activity in the parahippocampal cortex, among other areas**.

				Coordinates			*t*-stat
				*X*	*Y*	*Z*
Negative	Right	Temporal	Parahippocampal cortex	32	−40	−16	−5.15
			Fusiform gyrus	26	−64	−12	−6.24
	Left	Temporal	Inferior temporal lobe	−50	−52	−8	−5.29
			Fusiform gyrus	−34	−54	−10	−7.15
		Parietal	Parietal lobe	−32	−76	36	−5.48
		Occipital	Fusiform gyrus	−34	−54	−10	−7.15
			Middle occipital gyrus	−34	−78	22	−5.1
			Superior occipital gyrus	−28	−86	24	−4.96

*Brain areas showing changes in activity between the two baseline conditions. Positive values indicate an increase in fMRI activity in the second baseline condition, compared to the first baseline condition, for a specific set of stimuli. Negative values indicate a decrease in fMRI activity in the second baseline condition, compared to the first baseline condition, for a specific set of stimuli. Note that the spatial configuration task and the object identity task were not included in these analyses*.

#### Object Task

During the object task, there was activity in the anterior temporal lobes bilaterally (left: *x* = −46, *y* = 8, *z* = −34; *t* = −4.15, *p* < 0.005; right: *x* = 34, *y* = 0, *z* = −28; *t* = −3.99, *p* < 0.005; Figure 9, Table 2).

When contrasting the two baseline conditions, before and after the object task for that specific set of stimuli, there were decreases in activity in the left fusiform and RPH (Figure 10B; Table 4). Interestingly, however, the decrease in left fusiform cortex activity was specific to the object identity condition. This is interesting in the light of the fact that participants did not engage in any task during these baseline conditions and that the pattern of change that occurred in the contrast between these baseline conditions was different for the baseline conditions around the spatial task vs. those around the object task.

**Table 4:** **Contrast of baseline fMRI scans before and after the object task showing the decrease in fMRI activity in the fusiform gyrus, among other areas**.

				Coordinates			*t*-stat
				*X*	*Y*	*Z*
Positive	Right	Parietal	Parietal lobe	48	−52	34	6.15
	Left	Frontal	Precentral gyrus	−66	−6	16	4.94
		Parietal	Cingulate gyrus	−4	−34	38	4.89
Negative	Right	Temporal	Parahippocampal cortex	26	−42	−10	−4.72
		Occipital	Middle occipital gyrus	34	−80	14	−6.55
			Inferior occipital gyrus	30	−88	4	−6.7
			Fusiform gyrus	42	−68	−10	−6.24
	Left	Temporal	Fusiform gyrus	−38	−40	−22	−5.64
		Occipital	Fusiform gyrus	−38	−48	−16	−6.34
			Middle occipital gyrus	−38	−84	14	−5.94

*Brain areas showing changes in activity between the two baseline conditions. Positive values indicate an increase in fMRI activity in the second baseline condition, compared to the first baseline condition, for a specific set of stimuli. Negative values indicate a decrease in fMRI activity in the second baseline condition, compared to the first baseline condition, for a specific set of stimuli. Note that the spatial configuration task and the object identity task were not included in these analyses*.

### Discussion

An increase in activity in the parahippocampal cortex was measured during the spatial task compared to the object task, thus supporting results obtained in the cognitive experiment discussed previously, showing that the parahippocampal cortex was critical for the spatial task in patients with specific lesions to the parahippocampal cortex. The behavioral response, task and visual presentation of the two baseline subtraction conditions were identical; yet, the spatial and object tasks administered between the two conditions modulated the activity of the parahippocampal cortex. In addition, the object task modulated the activity of the left fusiform gyrus more than the spatial task. Since patients with lesions to the RPH performed well on the object task, the left fusiform gyrus may have represented the information critical for the object identity task. These results provide further confirmation that the parahippocampal cortex is involved in acquisition and long-term changes related to the spatial configural task tested in the Oddball fashion.

The inferior parietal cortex was activated in healthy participants when they performed the spatial task in the fMRI scanner. Structurally and functionally, there are extensive connections between the parietal cortices and the parahippocampal cortex (Van Hoesen, 1982; Blatt et al., 2003). Increased connectivity between the parahippocampal cortex and parietal cortices (specifically, the angular gyrus) has been found during tasks that require the identification of novel objects (Howard et al., 2013). It is possible that the inferior parietal cortex plays a role in recognizing changes in the spatial layout of the scenes presented. Ciaramelli et al. (2010) showed that the parietal cortex was specifically involved in egocentric navigation by showing that patients with damage to the posterior parietal cortex were specifically impaired in an egocentric route learning task, while allocentric navigation was spared. In addition, the inferior parietal cortex may play a role in bringing attention to details of the environment (Cabeza et al., 2008). Berryhill et al. (2007) showed that patients with bilateral damage to the inferior parietal cortex exhibited a deficit in autobiographical memory. Specifically, there was a deficit in richness of details when the patients freely recalled autobiographical memories. In the present study, there was activity in the inferior parietal cortex when participants were presented with a new spatial configuration. The activity in the inferior parietal cortex may be due to the attentional demands required when there is a specific change in spatial configuration.

It is interesting to note that, since our object task and spatial task were similar in perceptual stimulation (perception of five objects on a screen), the significant fMRI activity observed in the parahippocampal cortex (during the spatial task minus object task, or the contrast between the two baseline conditions) was a reflection of top down processing (not perceptually driven by the five objects). In other words, the differential activity measured between the two identical baseline tasks or the two active experimental tasks are likely to have represented the information processing relative to the instructions and not relative to the perceptual representation of the stimuli, since these were similar across all conditions. The fact that in one case there was an increase in fMRI activity in the parahippocampal cortex (in the contrast between the two active conditions) and that in the other case there was a decrease in fMRI activity in the parahippocampal cortex (in the contrast between the two baseline conditions) suggest that the changes in fMRI activity to identical perceptual features do not reflect any kind of habituation to the stimuli. As such, we conclude that activity measured in the parahippocampal cortex reflected top down processing.

Furthermore, repetition suppression was previously reported in the literature when stimuli were repeatedly presented (Brozinsky et al., 2005; Epstein et al., 2007, 2008). While repetition suppression typically refers to very rapidly presented stimuli, and is thought to reflect specific neuronal effects (reduced firing on the second of two rapid stimuli; Epstein et al., 2001) our data show different patterns of repetition suppression for the different tasks. This provides strong evidence that repetition suppression can come as a result of learning. In the literature, repetition suppression was observed in the parahippocampal cortex (Epstein et al., 2005, 2007, 2008). In Epstein et al. (2005), it was found that good navigators showed larger repetition effects than bad navigators suggesting that better learning had an impact on suppression of fMRI activity. These data suggest that repetition suppression can implicate a reduction of activity that occurs with learning.

## General Discussion

The combined results from these two studies highlight the importance of the parahippocampal cortex in spatial configural learning, independently from the hippocampus. Functional brain imaging experiments have implicated the parahippocampal cortex in different tasks requiring spatial processing. For example spatial navigation in a virtual, visual maze produced extensive activations of the parahippocampal cortex, bilaterally, in both fMRI (Brewer et al., 1998) and PET (Maguire et al., 1996) studies. Viewing scenes has also activated the parahippocampal cortex in fMRI experiments (Stern et al., 1996; Brewer et al., 1998; Epstein and Kanwisher, 1998). Further, participants viewing a videotaped tour of houses displayed increased activation in the right parahippocampal gyrus when asked to recall spatial location or temporal order, but not object identity (Hayes et al., 2004). Interestingly though, it has been shown through a novel paradigm that the parahippocampal cortex is involved in the formation of contextual associations regardless of spatial information content (Aminoff et al., 2007).

A systematic study of the components of a scene that elicit activity in the parahippocampal cortex revealed that the walls forming an indoor room, without objects, were sufficient to produce high activity in the parahippocampal cortex (Epstein and Kanwisher, 1998) but that the spatial arrangement of objects or single objects was not sufficient for activation of the parahippocampal cortex. Interestingly, familiar landmarks, represented by buildings cut out from their background produced a signal in the parahippocampal cortex significantly higher than the response to household objects. This study strongly suggested that the parahippocampal cortex processes information related to places (Epstein and Kanwisher, 1998). In support of this, on an fMRI task, Howard et al. (2011) found that activity in the parahippocampal cortex was related to the presentation of novel scenes. This function was separate from the hippocampus, which showed more selective activity to changes in the spatial relationship between objects and their background context. However, there are also alternate findings in the literature supporting a theory that the parahippocampal cortex also mediates contextual associations (Bar et al., 2008). In Janzen and van Turennout (2004), participants navigated through an environment in which objects were places at navigationally relevant (serving as landmarks) and non-relevant locations. Later on, when the objects were presented on their own, outside of the environment, the parahippocampal cortex was active only for objects that were navigationally relevant, even when the participant did not consciously recollect seeing the object. Our study showed that the parahippocampal cortex was critical for memory for the configuration of objects even without a background. Perhaps the memory component for the spatial arrangement of objects in our task involved the parahippocampal cortex in a way not needed during passive viewing of objects. The findings suggesting that the parahippocampal cortex responds to a landmark (e.g., building) and not an object (e.g., blender) support the idea that the spatial attribute of the stimuli tapped into the function of the parahippocampal cortex.

In another study, the magnitude of the parahippocampal activation was correlated with subsequent recollection of single scenes in a single event fMRI study, thus implicating the parahippocampal cortex in memory for scenes (Brewer et al., 1998). Imaging studies implicating the parahippocampal cortex in memory for scenes and lesion studies that show a dissociation between the parahippocampal cortex and the hippocampus support the hypothesis that the parahippocampal cortex itself can sustain memory.

Several studies have noted the role of the retrosplenial cortex in spatial learning (Epstein and Higgins, 2007; Epstein et al., 2007; Auger et al., 2012; Auger and Maguire, 2013; Epstein and Vass, 2014). However, in the current paradigm, we did not observe activity in the retrosplenial cortex. There are several reasons that may explain this discrepancy, one of which may be the low sample size. However, findings from the current fMRI study are consistent with behavioral findings from patients with lesions tested on the same paradigm, thus providing validity to our results despite the low sample size. An alternate explanation may be related to the function of the retrosplenial cortex. Although the exact role of the retrosplenial cortex is unclear, in Epstein and Vass (2014), the restrosplenial cortex is described as playing a role in localization and orientation in an environment. It is also described as playing a role in coding stable landmarks in an environment and storing spatial knowledge between well-traveled locations. The current paradigm does not require localization, orientation, or coding of permanent landmarks in an environment. Therefore, for this reason, we may not have observed activity in the retrosplenial cortex.

The studies of patients with thermal lesions have shown that both the RH and the RPH are important in spatial memory (Bohbot et al., 1998). The RPH seems to be involved in memory for spatial configuration of objects as well as memory for a location in an environment found during real space navigation. The hippocampus, on the other hand, seems to be involved in forming spatial relationships between environmental objects or landmarks in order to construct cognitive maps used in navigation (Iaria et al., 2003; Konishi et al., 2013). For example, the object location task that was sensitive to selective lesions to the hippocampus, sparing the parahippocampal cortex, required patients to construct a top-down view of the environment from their first person view experience; hence they had to generate a cognitive map (Bohbot et al., 1998). When the first person view is sufficient for navigation (i.e., they do not need to construct a top-down view in order to navigate), the parahippocampal cortex seems to be sufficient (Bohbot et al., 1998). While the current task required building spatial relationships, it did not necessarily require the construction of a cognitive map because one could learn the spatial configuration of stimuli by memorizing the spatial relations in a single scene (Bohbot et al., 1998; Brewer et al., 1998; Epstein and Kanwisher, 1998). Furthermore, when tested in a dual solution task previously shown to be dependent on either the hippocampus or the caudate nucleus with fMRI (Iaria et al., 2003), patients with brain damage to the hippocampus are severely impaired at constructing cognitive maps (i.e., remembering the locations of target objects in relation to environmental landmarks; Bohbot et al., 2004). Interestingly, the parahippcampal cortex sends massive projections to both the hippocampus and caudate nucleus. Therefore, the parahippocampal cortex may provide the spatial information necessary for building stimulus-response relationships at the level of the caudate nucleus used for navigation (i.e., navigating by learning a sequence of specific motor behaviors such as a right or left turn, when reaching landmarks that act as stimuli; Packard et al., 1989; White and McDonald, 2002), and in parallel, spatial information from the parahippocampal cortex may be processed by the hippocampus to form cognitive maps. Further research targeting such dissociations is necessary to delineate the specific spatial roles played by the parahippocampal cortex and the hippocampus. It is interesting to note that, in rats, larger hippocampal lesions are required to cause object recognition deficits than spatial memory deficits (Broadbent et al., 2004), suggesting in a different way, that the hippocampus is more critical for spatial memory than object recognition. The present results show that the parahippocampal cortex itself plays a critical role in spatial memory that can be dissociated from that of the hippocampus. The present experiment is also the first to reveal task specific repetition suppression associated with repetition of the baseline control condition in the very same region proven to be critical for learning the task in a brain-lesion study. The latter findings suggest a top-down, task specific modulation of repetition suppression in the parahippocampal cortex.

## Author Contributions

Experimental design of the oddball task by JJBA and LN, experimental design of the fMRI study by SOD, VDB, AD, MP and ACE. The neurological examination of the patient study was performed by MK. Data collection of the patient study was performed by VDB and KS and for the fMRI study by VDB and SOD. VDB, MP, JJBA and LN wrote the manuscript.

## Conflict of Interest Statement

The authors declare that the research was conducted in the absence of any commercial or financial relationships that could be construed as a potential conflict of interest.
